# Reproductive Parameters in the Critically Endangered Blue-Throated Macaw: Limits to the Recovery of a Parrot under Intensive Management

**DOI:** 10.1371/journal.pone.0099941

**Published:** 2014-06-18

**Authors:** Igor Berkunsky, Gonzalo Daniele, Federico P. Kacoliris, José A. Díaz-Luque, Carmen P. Silva Frias, Rosana M. Aramburu, James D. Gilardi

**Affiliations:** 1 Instituto Multidisciplinario sobre Ecosistemas y Desarrollo Sustentable, Universidad Nacional del Centro de la Provincia de Buenos Aires, Tandil, Buenos Aires, Argentina; 2 Proyecto de Conservación de la Paraba Barba Azul, The World Parrot Trust, Sachojere, Beni, Bolivia; 3 División Zoología de Vertebrados del Museo de La Plata, Universidad Nacional de La Plata, La Plata, Buenos Aires, Argentina; 4 Instituto de Ciencias Ambientales y Evolutivas, Facultad de Ciencias, Universidad Austral de Chile, Valdivia, Chile; 5 The World Parrot Trust, Lake Alfred, Florida, United States of America; Behavioural Ecology & Ecophysiology group, Denmark

## Abstract

Rediscovered in the wild twenty years ago, the breeding biology of wild Blue-throated Macaws remains largely unexplored, yet is essential to its effective conservation and recovery. Here, we analyse reproductive parameters in an intensively managed wild population of Blue-throated Macaws, providing the first data on the breeding biology of this critically endangered species. During the six-year study period, 2007–2012, the number of active breeding pairs either remained constant or decreased, depending on the site, and no new breeding pairs were discovered despite extensive searching. We documented nesting attempts in natural cavities in dead palms or live hardwoods, and artificial nest boxes. Egg-laying was concentrated during the end of dry season and the beginning of the wet season, August through December. Hatching failure was the greatest cause of egg losses. Half of the breeding attempts of Blue-throated Macaws produced at least one fledging, on average two, after a 85 days nestling period. An average of 4.3 nestlings per year fledged from all known wild nests combined. Each pair lost roughly 65% of its initial reproductive investment at each nesting attempt. In most successful nesting attempts of individualized pairs, a new nesting attempt was not detected the following year. All monitored breeding pairs showed high nest site fidelity, reusing hardwood-tree cavities and nest boxes. Our findings will aid conservation efforts by refining current actions and prompting new approaches towards the conservation and recovery of the Blue-throated Macaw.

## Introduction

Nearly half of the 152 species of Neotropical parrots are threatened or near-threatened with extinction, and most of the remaining species are declining due to exploitation for the pet trade, hunting for food and feathers, and/or habitat destruction [1]–[3]. Although extensive parrot research and conservation work is on-going in the Neotropics, we still lack basic biological data for many taxa, hampering the identification of specific threats, the effective monitoring of populations, and the implementation of effective conservation actions.

Macaws are the most endangered group of the Psittacidae family with at least five extinct species, three critically endangered (one possibly extinct [*Anodorhynchus glaucus*], and one species [*Cyanopsitta spixii*] surviving only in captivity), and seven of the 15 species that remain in the wild occur on the Red List as Endangered, Vulnerable, or Near Threatened [3], [4]. The Blue-throated Macaw *Ara glaucogularis* is an Bolivian endemic, and one of the two critically endangered macaw species that still exists in the wild (as *A*. *glaucus* is possibly extinct and *C. spixii* can only be found in captivity) [5], [6]. In the wild, this macaw is unlikely to number more than 115–125 individuals divided in two subpopulations [7]. A number of conservation actions aimed to recover the wild population of Blue-throated Macaw were conducted during the last 10 years; however our knowledge of the species' biology is limited to descriptions and estimations of range, habitat use and population size [6], [8]–[10]. Basic reproductive parameters for wild Blue-throated Macaws remain unavailable, yet are fundamental to their conservation and recovery.

Here, we analyze a variety of reproductive parameters such as clutch size, hatching success, fledging success and nest success, in an intensively managed wild population of Blue throated Macaws. Our research provides the first published data about the breeding biology of this critically endangered species. Our observations were made concurrently to our intensive hands-on conservation program, with the primary purpose of maximizing these same reproductive parameters. Our findings will aid conservation efforts by refining current actions and prompting new approaches towards the conservation and recovery of the Blue-throated Macaw and other parrots facing similar threats.

## Methods

### Study site

**Figure 1:**
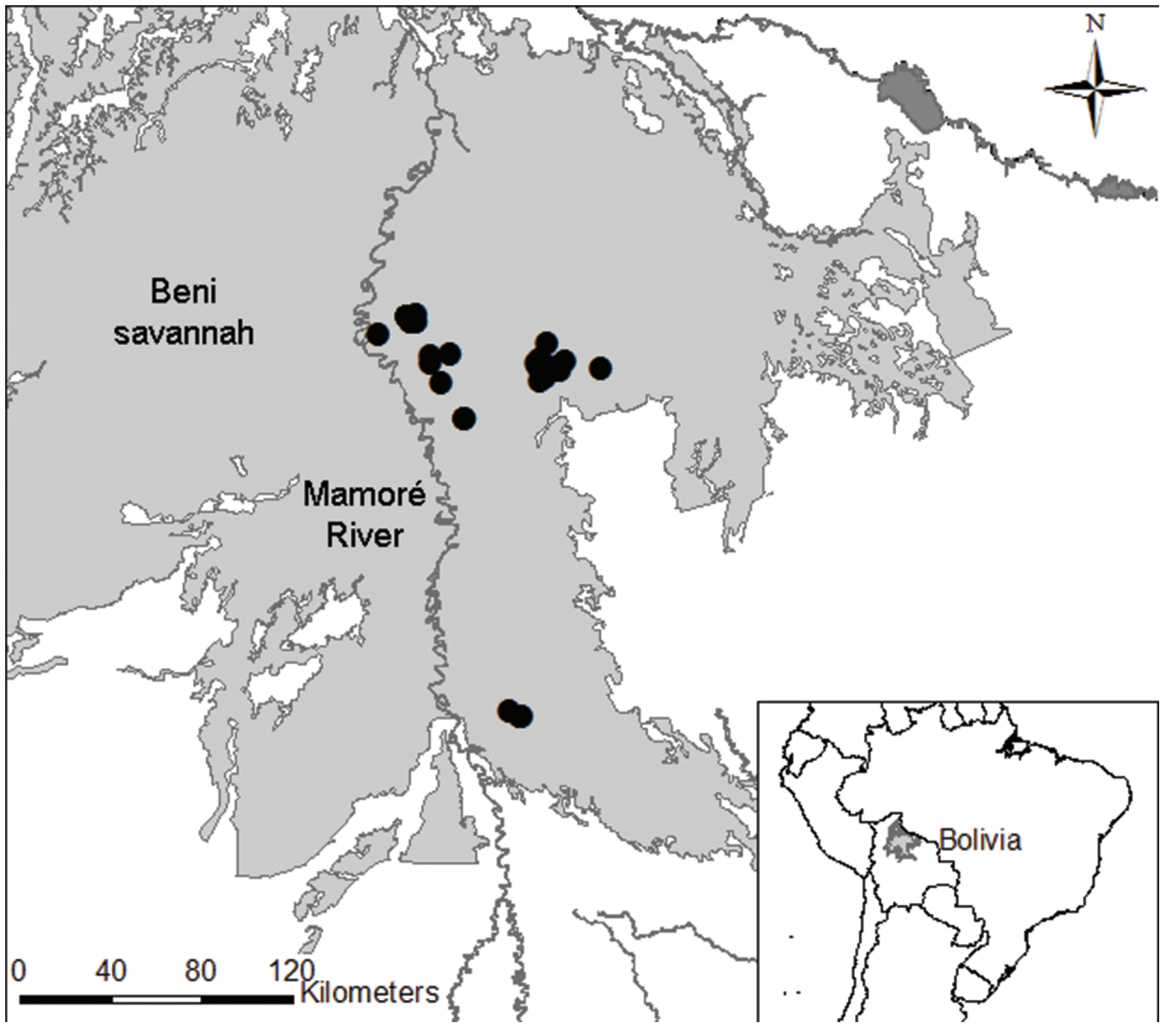
Location of active breeding pairs. Map showing all active breeding pairs of Blue-throated Macaw (black dots) during the 2007–2012 period.

We conducted surveys in the Llanos de Moxos, Beni Department, northern Bolivia (Fig. 1). The Llanos de Moxos is a 160,000 km^2^ expanse of seasonally inundated savannahs, interspersed with a complex mosaic of forest islands and riverine gallery forests, occupying the extremely flat Beni–Mamoré–Iténez basin in southwest Amazonia, situated between the Precambrian Shield to the east and the Andes to the west and south [11]. Numerous white-water rivers and hundreds of shallow, flat-bottomed lakes cover the landscape. Mean annual precipitation varies from 1,300 to 2,000 mm across the region, occurring mostly between September and May [12].

The landscape is dominated by flat, low-lying areas, which are seasonally inundated and covered by completely open, treeless savannah [13]. Conversely, forest islands are scarce and restricted to raised areas (mounds), which are sufficiently elevated to escape annual flooding. Most of forest islands are eroded relics of natural levees or terraces of abandoned river channels, and therefore constitute fragments of former gallery forest [14]. The study region maintains a high diversity and abundance of parrots, including large macaws (*Ara choropterus* and *Ara ararauna*) [8]. Most parrot populations in the area appear healthy, with the Blue-throated Macaw being the only highly threatened species.

Human presence in the study area occurs at low densities (1.4 people per square kilometre), with 43 settlements spanning the municipalities of Trinidad, San Javier, San Ramón, Santa Ana de Yacuma, San Andrés and Loreto. The primary occupation of the residents is cattle ranching [11] as has been the case for several hundred years.

### Data collection

Data were collected from early August up to late March during five consecutive breeding seasons (2007–2008 to 2011–2012). Each breeding season we searched intensively for Blue-throated Macaw nests throughout the season, visiting all known nesting areas for the species covering approximately 5200 Km^2^ of the Beni. We identified some individuals by photographing their distinctive facial feather tracts (enabling confirmation of site fidelity). We found nests mainly by observing the behaviour of breeding pairs, and after locating a potential nest (a tree with a cavity and macaw activity) we reached the entrance hole using modified single-rope ascending techniques [15]. For each nest we noted the following: tree species, diameter at breast height (DBH), diameter at entrance-hole height (DEH), height of the entrance hole, minimum and maximum diameter of the entrance hole, internal diameter and depth of the cavity.

We assigned a start date to each nest based upon the date of the first egg was laid in each nest. We defined as nesting attempt when at least one egg was laid in a nest. The start date of nests found before or during the laying stage was determined directly. The start date of nests found during incubation or nestling stages was determined by back-counting from the hatching date of the first egg and assuming a 25-day incubation (based upon eggs of known lay and hatch dates). We estimated the duration of the laying stage as the number of days between the first and the last laying event of the season.

In some cases, nests were observed from blinds between 25 and 40 m from nest trees. Daily observations were concentrated during the morning (dawn to 10:00) and the afternoon (15:00 to dusk). The frequency of nest visits by adult macaws was variable and it was related to the nest location and nest stage. For monitored nests, we climbed trees once a week during incubation; every day or every other day during the first three weeks of nestling development; and twice a week until the nestlings fledged. We recorded nest content each time we inspected a nest.

For each nest, we defined the duration of 1) the incubation period as the difference in days between the laying date and the hatching date of the last egg and 2) nestling period as the difference in days between the hatching date and the nest abandon date of the first nestling [16].

In order to calculate clutch and brood sizes we counted eggs and nestlings at four stages of the breeding period: a) at the end of the laying period (Total clutch laid), b) at the end of the incubation (Clutch size at hatching), c) immediately after the hatching (Brood size hatched) and d) immediately before nestlings' fledge (Brood size at fledgling). For each nest we also estimated the egg survival, as the proportion of eggs that complete the incubation period; the hatching success, as the proportion of the eggs at hatching that produced nestlings; and the nestling survival, as the proportion of the hatched nestlings that successfully fledged from the nest (i.e. fledglings). A nest was deemed successful if it produced at least one fledgling.

When loses occurred, we recorded the most probable cause as: a) predation (i.e. missing or broken eggs without any apparent cause, or eggs or nestlings gone from the nest with egg shells or feathers in the nest interior); b) unknown disease (dead nestlings in the nest without any external sign that allowed us to determine the cause of death), c) failure during hatching (eggs that had not hatched one week to ten days after the expected hatching date), d) adverse weather (e.g. a flooded cavity, a cavity broken open by wind), e) nest abandoned during incubation, f) nest poaching and g) starvation. Most unhatched eggs were left inside the nests. In some cases, we removed those eggs and we examined the egg content.

Fledging was confirmed by at least two of the following criteria: fully feathered young seen in the nest; new fledglings seen or heard nearby; nest undisturbed and in good condition in combination with one other of the criteria. Descriptive statistics are presented as averages ± SE. We performed all statistical analyses with significance accepted at P<0.05.

Observations were made concurrent to our intensive conservation program, which included a range of actions designed to enhance nesting success. 25 hardwood and 12 PVC nest boxes (for details see [17], [18]) were installed at sites where they were likely to be adopted by known pairs. We protected most occupied nest trees each year from predators with metal flashing, by pruning branches, and by actively defending nest sites from blinds during daylight hours. We manipulated tree cavities to keep them dry and safe by redirecting water away from the cavity entrance, placing drains in the cavity, or both. Finally, in some cases nestlings received supplemental food until their growth rates caught up with age-appropriate levels. The study involved a critically endangered and protected species and our field protocol was approved by the Dirección General de Biodiversidad, Viceministerio de Medio Ambiente of Bolivia (Permit Number: 1239-11, Project Name: Proyecto de Conservación de la Paraba Barba Azul: Manejo poblacional). The study was carried out on private lands (owner names are mentioned in acknowledgments).

## Results

During the 2007–2012 period we identified 64 individuals in the study area, of which at least 32 were active breeding birds, specifically 16 distinct pairs that laid at least one egg (Fig. 1). During our study, the number of active breeding pairs in a given area either remained constant or decreased, depending on the site.

We followed 31 nesting attempts (n = 12 in 2007–2008, n = 2 in 2008–2009, n = 8 in 2009–2010, n = 4 in 2010–2011, and n = 5 in 2011–2012). These nesting attempts occurred in 19 different natural cavities and six wooden nest boxes and one PVC nest box. Twelve natural cavities were in dead palms: 11 in *Attalea phalerata* and one in *Acrocomia aculeata*; and six in live hardwood trees: four *Gallesia integrifolia*, two *Anaedanthera colubrina;* and one in *Sterculia apetala*. Table 1 shows tree and cavity characteristics. Most cavities in hardwood trees and in nest-boxes were reused by Blue-throated Macaws at least once during the study period, while cavities in dead palms were never reused due to rapid degradation. All pairs that accepted nest boxes had nested in the same tree or in a tree no greater than a few meters from where the pair had bred in previous seasons.

**Table 1:** Main characteristics (mean ± SE) of natural cavities used as nest by Blue-throated Macaws.

Tree and cavity characteristics	Mean ± SE (n)	Range
DBH (cm)	65.7±12.2 (7)	30–107
Diameter at entrance (cm)	57.5±2.1 (4)	52–62
Height of entrance hole (m)	8.60±0.79 (17)	2.5–14.0
Depth of cavity (cm)	52.3±7.9 (11)	25–110
Maximum diameter of entrance hole (cm)	24.1±2.5 (8)	13–33
Minimum diameter of entrance hole (cm)	17.9±3.6 (8)	9–33
Internal diameter of cavity (cm)	28.8±1.0 (6)	26–33

Sample sizes (number of trees) are indicated between parentheses.

**Figure 2:**
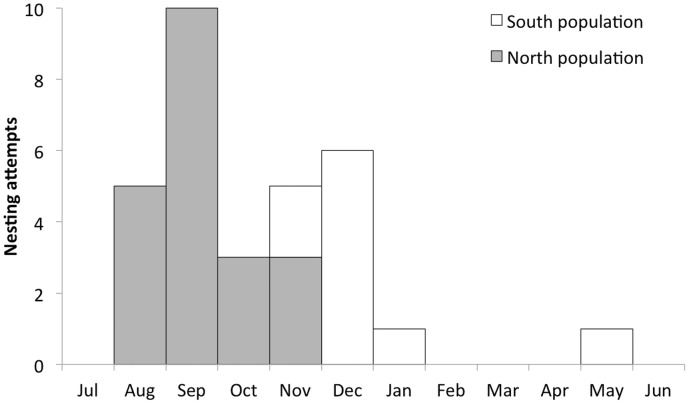
Clutch initiation. Phenology of clutch initiation for 31 nesting attempts of Blue-throated Macaw during five consecutive breeding seasons (2007–2008 to 2011–2012).

Egg-laying was generally concentrated during the end of dry season (i.e. September and October) in the northern population and during the beginning of the wet season in the southern population, resulting in a large laying interval from August to May (Fig. 2).

The average clutch size was 2.53±0.10 eggs per clutch (*n* = 29), with a range of one to three and a mode of three eggs per clutch. Clutch replacement was observed twice and only in nests that failed during incubation. No eggs from second clutches hatched.

The precise duration of incubation was clearly documented for one nest and lasted 25 days for each egg. During egg-laying and incubation, females spent most of their time inside the nest or perched nearby, and males were rarely seen entering the nest cavity, although they often remained perched nearby. Partial losses during incubation were low: two cracked eggs were removed by parents before hatching date.

The average clutch size at hatching was 2.10±0.18 eggs per clutch (range: 1–3; *n* = 21). Hatching success was 72±7% (*n* = 23) and hatching failure was the greatest cause of egg losses (28/30 eggs). In most nests all eggs hatched (52%, *n* = 12), and in some nests one egg (30%, *n* = 9) failed to hatch and in two nest none of eggs hatched (9%, *n* = 2). Two of four (50%) of non-hatched eggs that were removed had a partially developed embryo. The average date of hatch for the first nestling in a nest was October 23^th^ (from May 3^rd^ to January 15^th^, *n* = 21).

The average clutch size at fledgling was 2.00±0.25 fledglings per clutch (range: 1–3; *n* = 13 nests). In successful nests, the survival of nestlings was 100% (*n* = 25 nestlings).

The nestling period lasted approximately three months (85 days).

If we pool all nests, of 74 eggs (n = 29 nests), 30 eggs were lost during the incubation period and 18 nestlings were lost during the nestling rearing period (59% of overall survival of hatchlings). Between 2007 and 2012, a total of 26 nestlings of Blue-throated Macaw successfully fledged, meaning an average of 4.3 nestlings per year fledged from all known nests during the study period. Given that the average total clutch was 2.5 eggs per nesting attempt, and the average of 0.89 fledglings per nesting attempt, each pair is losing on average 65% of its initial reproductive investment at each nesting attempt.

From 30 breeding attempts of Blue-throated Macaws, 77% of theses (*n* = 23) would complete the incubation stage and 45% (*n* = 13) would produce at least one fledging (i.e. nesting success).

**Figure 3:**
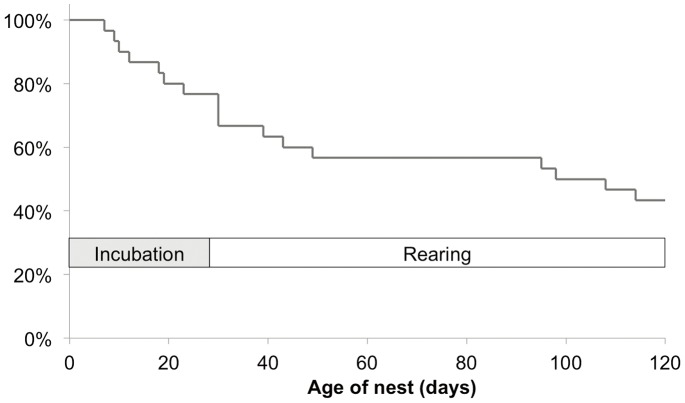
Nest survival curve. Kaplan-Meir survival curve for Blue-throated Macaw nests (*N* = 30 nesting attempts) in Beni savannahs.

Fifty-seven per cent of 30 monitored nests failed. Most failures occurred during the incubation stage (Fig.3). Causes of nest failure were diverse (Table 2). All evidence of predator identity was indirect. In two cases the evidence suggested predation by snakes. An unknown disease appears to be affecting breeding success in one breeding pair and was responsible for the death of all nestlings in the clutch, in three nesting attempts in sequential years. Nest abandonment as a result of adverse weather included nest-tree falls and broken and flooded cavities.

**Table 2:** Failed nesting attempts and causes of nest failure of Blue-throated Macaw pairs per breeding season.

Cause of nest failure	Breeding season				
	2007-08 (12)	2008-09 (2)	2009-10 (8)	2010-11 (4)	2011-12 (5)
Predated	4	1	-	-	-
Apparent disease	-	1	1	-	1
Failure to hatch	1	-	1	-	-
Adverse weather	2	-	-	-	-
Abandoned	-	-	-	1	1
Poached	-	-	2	1	-

In parenthesis the number of nesting attempts.

In seven of eight successful nesting attempts of individualized pairs, a new nesting attempt was not detected the following year (Table 3). All monitored breeding pairs showed a high nest site fidelity reusing hardwood-tree cavities and nest boxes.

**Table 3:** Breeding attempt histories of seven Blue-throated Macaw pairs.

Pair	2007	2008	2009	2010	2011
A	S	NN	S	NN	S
B	S	NN	S	NN	Fa
C	Fp	NN	S	NN	S
D	1^ST^ Fw - 2^nd^ Fp	NN	S	NN	U
E	1^ST^ Fp - 2^nd^ Fh	NN	Fh	NN	U
F	S	Fp	-	-	-
G	U	Fu	Fu	Fa	Fu

S (successful), NN (no nesting attempt detected), U (unknown fate) and F (failed nest). Causes of failure were: Fa (failed by nest abandon), Fh (failed by hatching failure), Fp (failed by predation), Fu (Nestling's die or did not fledge for undiagnosed reasons) and Fw (failed by adverse weather).

## Discussion

During the study period, no new adult pairs were recruited into the breeding population. This lack of recruitment of breeding pairs could be a consequence of a low survival rate of juveniles and pre-breeding birds, and/or an extremely low density impeding effective pair formation. We have no information about survival of pre-breeding birds, and the density of breeding pairs is of 0.003 pairs/Km^2^ (i.e. 16 breeding pairs in 5200 km^2^). Failure to recruit new breeding pairs into the population, will of course severely constrain the potential for this critically endangered species to once again thrive in the wild.

Clutch sizes were similar to reported values in captive and wild pairs where the mode of three eggs is also common [18], [19], [21], [22]. The average clutch size for wild large macaws (*A. macao*, *A. chloropterus*, and *A. ararauna*; body mass 1015–1250 g) is 2.5–2.8, slightly higher than the average of 2.1 reported for the Blue-throated Macaw in this study [17], [18]. Hatching success of Blue –throated Macaw (72%) was similar to other macaw species reported from Peruvian rainforest where usually one egg does not hatch; 77% of hatching success in Red-and-Green Macaw (*Ara chloropterus*) and 50% in Scarlet Macaw (*Ara macao*); and considerably higher than the 36% of hatching success reported in Blue-and-Yellow Macaw (*Ara ararauna*) [17], [18]. In addition, as was found in large macaws of Peruvian rainforest, all eggs hatched in 61% of occupied nests [18]. Because inbreeding depression (or loss of heterozygosis) in birds is often indicated by depressed hatching success, our observations suggest that these birds have not yet suffered a significant genetic bottleneck, presumably because their population decline was so recent [21].

Clutch replacement was not observed in nests that failed during the rearing of nestlings. We observed a replacement clutch only on two occasions, and in both cases nests failed during first days of incubation. Second clutches are not common in wild parrots, in fact, in macaws they have been documented only in Scarlet Macaws [15], [22], [23]. The decision of some pairs of macaws to initiate a second clutch may be related to good body condition of the female or abundant food during the breeding period, or both.

Nesting attempts and nesting success varied between years. In some years, up to 10 pairs nested and in some years only two attempts were made. Nesting success of Blue-throated Macaw (45%) was similar to other macaw species where usually half of nesting attempts succeed: 54% in Blue-and-Yellow Macaws (*Ara ararauna*), 44% in Scarlet Macaws (*Ara macao*) and 41% in Red-and-Green Macaws (*Ara chloropterus*); but our value was lower than the 70% reported for Hyacinth Macaws (*Anodorhynchus hyacinthinus*) [18], [24], [25]. However, the number of fledgling per initiated nest (0.89) in Blue-throats was higher than the reported values for macaw species in Peruvian rainforest (between 0.55 and 0.65) and similar to the 0.98 reported value in Hyacinth Macaws [24], [25]. Differences in those indices are a consequence of the number of fledglings per successful nest. We observed two fledglings per successful pair, a value 38% higher than the maximum reported value for a large macaw in the wild (i.e. 1.44 fledglings/successful nest in *Ara macao*) [18]. This high nestling survival (100%) in successful pairs is probably a consequence of our intensive conservation management program.

Two thirds of natural cavities were in dead palms. Most cavities in hardwood trees were reused by Blue-throated Macaws at least once during the study period, while cavities in dead palms of *Attalea phalerata* were never reused before they fell naturally. Like other macaws [17], [18], [26], Blue-throats were willing to nest in various kinds of nest boxes, but in all cases, they selected boxes placed in the same tree or in a tree few meters from where the pair had bred in previous seasons.

We now have a better idea about the characteristics of cavities selected by Blue-throated Macaw to breed. Our findings on the dimensions suggested nothing unusual about these macaws, but the results are useful for future nest box design, which can be more carefully tailored to the species' needs and preferences. Future studies of nest site selection should assess the availability of similar cavities in the region.

Our results suggest that successful breeding pairs are unlikely to breed the following year; if true, this factor dramatically constrains this species' ability to recover from its current critically endangered status. In some years we observed breeding pairs accompanied with their fledglings of previous year, and they were not showing breeding behavior. Parents appear to attend to their fledglings (i.e. providing food, social learning, etc.) for an extended period, possibly through the subsequent breeding season in some cases. Further studies are needed to understand post-breeding relationships between adults and their dependent juveniles. This factor has potentially dramatic consequences for the reproductive output of the most productive and attentive breeding pairs. Our data also suggest that breeding pairs that failed in a given year are unlikely to make a breeding attempt the following year, or in some cases they may have separated and/or moved to different undiscovered breeding areas. Expectations of population dynamics and recovery potential for the species must integrate these natural limitations to be accurate and useful for conservation planning.

We fully recognize there are trade-offs to conducting research and applied conservation simultaneously on the same individuals in the wild. For a critically endangered species like the Blue-throated Macaw, with typically fewer than ten pairs attempting to breed in a given year, we feel a balanced approach putting conservation first, data gathering second, is appropriate. This approach has aided the species recovery while simultaneously generating biological findings, which inform ongoing and future conservation and management options. We cannot know how unmanaged populations would fare in terms of nesting attempts, rates of predation, hatching success, and recruitment of fledglings as all such parameters have been directly influenced by our actions. As this macaw's numbers improve in future years, we may have the opportunity to make direct comparisons between managed and unmanaged pairs, but with annual recruitment still averaging in the single digits, we do not yet have the luxury of taking such a hands-off approach.
